# Machine Learning Risk Prediction for Incident Heart Failure in Patients With Atrial Fibrillation

**DOI:** 10.1016/j.jacasi.2022.07.007

**Published:** 2022-11-01

**Authors:** Yasuhiro Hamatani, Hidehisa Nishi, Moritake Iguchi, Masahiro Esato, Hikari Tsuji, Hiromichi Wada, Koji Hasegawa, Hisashi Ogawa, Mitsuru Abe, Shunichi Fukuda, Masaharu Akao, Fushimi AF Registry Investigators

**Affiliations:** a Department of Cardiology, National Hospital Organization Kyoto Medical Center, Kyoto, Japan; b Division of Neurosurgery, St. Michael’s Hospital, Toronto, Canada; c Department of Arrhythmia, Ogaki Tokushukai Hospital, Gifu, Japan; d Tsuji Clinic, Kyoto, Japan; e Division of Translational Research, National Hospital Organization Kyoto Medical Center, Kyoto, Japan; f Department of Neurosurgery, National Hospital Organization Kyoto Medical Center, Kyoto, Japan

**Keywords:** atrial fibrillation, heart failure, machine learning, risk prediction, AF, atrial fibrillation, AUC, area under the receiver operating characteristics curve, HF, heart failure, LV, left ventricular, ML, machine learning, SHAP, Shapley Additive exPlanation

## Abstract

**Background:**

Atrial fibrillation (AF) increases the risk of heart failure (HF); however, little focus is placed on the risk stratification for, and prevention of, incident HF in patients with AF.

**Objectives:**

This study aimed to construct and validate a machine learning (ML) prediction model for HF hospitalization in patients with AF.

**Methods:**

The Fushimi AF Registry is a community-based prospective survey of patients with AF in Fushimi-ku, Kyoto, Japan. We divided the data set of the registry into derivation (n = 2,383) and validation (n = 2,011) cohorts. An ML model was built to predict the incidence of HF hospitalization using the derivation cohort, and predictive ability was examined using the validation cohort.

**Results:**

HF hospitalization occurred in 606 patients (14%) during a median follow-up period of 4.4 years in the entire registry. Data of transthoracic echocardiography and biomarkers were frequently nominated as important predictive variables across all 6 ML models. The ML model based on a random forest algorithm using 7 variables (age, history of HF, creatinine clearance, cardiothoracic ratio on x-ray, left ventricular [LV] ejection fraction, LV end-systolic diameter, and LV asynergy) had high prediction performance (area under the receiver operating characteristics curve [AUC]: 0.75) and was significantly superior to the Framingham HF risk model (AUC: 0.67; *P* < 0.001). Based on Kaplan-Meier curves, the ML model could stratify the risk of HF hospitalization during the follow-up period (log-rank; *P* < 0.001).

**Conclusions:**

The ML model revealed important predictors and helped us to stratify the risk of HF, providing opportunities for the prevention of HF in patients with AF.

Atrial fibrillation (AF) is the most common cardiac arrhythmia in the aging society and is associated with significant mortality and morbidity.^1^ Although thromboembolism is a well-recognized and preventable complication of AF, the incidence of heart failure (HF) remains high and is now more common than thromboembolism in these patients.^2^^,^^3^ In addition, HF accounts for a substantial proportion of deaths in contemporary patients with AF.^4^^,^^5^ However, many studies have focused on the prevention of thromboembolism, and little attention has been placed on the risk stratification for, and prevention of, HF despite its high prevalence and poor prognostic impact in patients with AF. An important step toward HF prevention is to identify patients who have a high risk for the disease. Therefore, comprehensive risk stratification of incident HF is warranted for the management of AF in daily practice; however, there is a scarcity of published reports regarding these issues.

Machine learning (ML) is a subset of artificial intelligence in which algorithms learn from data without explicit programming. ML techniques provide a powerful tool for learning complex relationships between the risk predictors and clinical outcomes from a representative sample of patients.^6^^,^^7^ Besides, ML can efficiently process huge multicategorical data, including biological, clinical, and imaging data, to predict the clinical outcomes.^8^^,^^9^ Recent studies, including ours, revealed that ML models can achieve higher prediction performance for thromboembolism than the validated risk score, the CHA_2_DS_2_-VASc score, in patients with AF.^10^, ^11^, ^12^ We consider ML techniques promising for risk prediction of future HF events; however, risk stratification for HF using ML algorithms in patients with AF has not been investigated.

Accordingly, the aim of the present study was to construct an ML model for predicting the incidence of HF events and to validate its performance using the data from a large-scale community-based prospective survey of Japanese AF patients, the Fushimi AF Registry.

## Methods

### Data source

The Fushimi AF Registry is a community-based multicenter prospective observational survey of patients with AF who visited the participating medical institutions in Fushimi-ku, Kyoto, Japan. The detailed study design, patient enrollment, and definition of the measurements of the registry were previously described (UMIN000005834).^13^^,^^14^ Briefly, the inclusion criterion for the registry is the documentation of AF on 12-lead electrocardiography or Holter monitoring at any time. There were no exclusion criteria. A total of 81 institutions, all of which are members of the Fushimi Medical Association, participated in the registry. The participating institutions comprised 2 cardiovascular centers, 10 small and medium-sized hospitals, and 69 primary care clinics. We started to enroll patients in March 2011, and enrollment ended in May 2017. All of the participating institutions attempted to enroll all consecutive patients with AF under regular outpatient care or admission. Collection of the follow-up information was mainly conducted through review of the medical records, and additional follow-up information was collected through contact with patients, relatives, and/or referring physicians by mail or telephone. Data of the patients were registered in the Internet Database System by the doctors in charge at each institution. Data were automatically checked for missing or contradictory entries and values out of the normal range. Additional editing and checks for duplicated records were performed by clinical research coordinators at the general office of the registry. The study protocol conformed to the ethical guidelines of the 1975 Declaration of Helsinki and was approved by the ethical committees of the National Hospital Organization Kyoto Medical Center (10-058) and Ijinkai Takeda Hospital (14-033).

### Outcomes

The primary endpoint in this study was the incidences of hospitalization for HF during the follow-up period. HF hospitalization was determined by the attending physicians based on history, clinical presentation (symptoms and physical examinations), natriuretic peptide levels, imaging findings including chest x-ray and echocardiography, cardiac catheterization findings, response to HF therapy, and in-hospital course. We continued follow-up until the death endpoint, and we defined clinical outcomes as the time to first event.

### Data processing

For the purpose of creating and validating the ML model, we divided the entire cohort of the registry into a derivation cohort and validation cohort. The data of patients from 1 cardiovascular center and half of the small and medium-sized hospitals and primary care clinics, which were randomly selected, were assigned as the derivation cohort. The data of patients from the other cardiovascular center and the remaining half of hospitals and primary care clinics were assigned as the validation cohort.

The data included baseline patient characteristics, oral prescription, the results of blood tests, and imaging data derived from chest x-ray and transthoracic echocardiography at registration. A total of 168 baseline variables were included in the data set of the Fushimi AF Registry. For data preprocessing, variables that were not clinically meaningful (for example, enrollment date) were deleted at the investigators’ discretion based on the clinical perspective. In addition, several variables were created using existing variables (for example, body mass index was calculated using patients’ height and weight data). Variables with more than 30% missing data in the derivation cohort were deleted.^12^ We did not include medication history in the ML model, considering the difficulty in interpreting the cause–effect relationship. Finally, 66 variables were listed as candidates for constructing the ML model (Supplemental Table 1). As was applied in the previous studies and ours,^12^^,^^15^^,^^16^ the missing values were imputed using the mean value for continuous variables, and using the mode for dichotomous variables from the derivation cohort.

### Model derivation

Supervised ML was used. The specific model algorithms used in this study were random forest, light gradient boosting machine, elastic net, linear support vector machine, neural network, and naive Bayes model. All of these 6 ML algorithms are fully established and commonly used for artificial intelligence prediction tasks. For the model derivation, including training and hyperparameter tuning, and internal evaluation, we performed 5-fold cross-validation in the derivation cohort. In the training step, model hyperparameters were optimized with a grid search algorithm. Grid search tunes and optimizes the model hyperparameters using a greedy way (the actual hyperparameters are shown in Supplemental Table 2). To evaluate the performance of the ML model, the sensitivity, specificity, accuracy, and area under the receiver operating characteristics curve (AUC) were evaluated for each algorithm. To explain which variables the model mainly relied on to arrive at a final prediction, the importance of each variable was calculated as the Shapley Additive exPlanation (SHAP) value.^17^ The SHAP value estimates each variable’s contribution based on cooperative game theory. When calculating SHAP values, an ML model is approximated with a simple model in which the contribution of each variable is easily explained and the degree of contribution is calculated as the SHAP value. The model algorithms, cross-validation, and grid search were based on the Python library scikit-learn.

### Final variable selection and validation

After the model derivation, each of 6 ML models was evaluated for its performance using the validation cohort. For model evaluation with the validation cohort, the missing values were imputed 20 times with multiple imputation with chained equations to address the randomness of the estimation.^18^^,^^19^ In the imputation process, all other variables in the validation cohort were used to create imputed results. Model performance metrics were displayed with 95% confidence intervals using 2,000 iterations of bootstrap.

Subsequently, we created a practical ML model using several variables with a view of future applicability. Inasmuch as there are no clear criteria for variable selection, we extracted several variables based on validity, feasibility, and applicability from the clinician’s perspective. Considering the SHAP value and the comparable predictive ability across each ML model, we finally selected 7 variables in the random forest algorithm for the practical ML model. Then, 6 ML algorithms using these 7 variables were also evaluated for performance using the validation cohort. Thereafter, we compared the receiver operating characteristics curve based on the random forest algorithm using 7 variables with that of the Framingham HF risk model.^20^ Given that there is no validated risk model for predicting incident HF among patients with AF, we adopted the Framingham HF risk model, which is considered to be the most famous HF risk model for patients with cardiovascular diseases. We defined left ventricular (LV) hypertrophy as interventricular septum thickness ≥12 mm instead of the electrocardiogram criteria in the original model.^20^ The Kaplan-Meier curves were plotted to display the clinical course among the subgroups stratified by the random forest algorithm using 7 variables in the validation cohort. The distribution of the predicted probability was divided into tertiles: low risk was defined as probability in the first tertile, intermediate risk as probability in the second tertile, and high risk as probability in the top tertile. Last, we specifically examined the predictive performance of practical ML models among patients without pre-existing HF in the validation cohort.

### Statistical analysis

Continuous variables are presented as the mean ± SD when normally distributed, and as the median and interquartile range when non-normally distributed. Distribution was assessed using histograms. The Kaplan-Meier method was used to estimate the cumulative incidences of clinical outcomes, and log-rank testing was performed to assess differences among groups. The hazard ratio of the events was calculated using the Cox proportional hazards model. Receiver operating characteristics curves were compared using the Henley and McNeil method.^21^ All statistical tests were 2-tailed, and a value of *P* < 0.05 was considered significant. All analyses were performed using JMP version 14.2.0 (SAS Institute) and R statistical software version 4.0.0.

## Results

### Baseline characteristics

We obtained a total of 4,396 patients with follow-up data by April 2019. We excluded 2 patients without the data of HF hospitalization during the follow-up period. Of the 4,394 patients, the mean age was 73.6 ± 10.9 years, and 1,765 (40%) were female. Paroxysmal AF accounted for 2,194 (50%) patients, and 1,204 (27%) patients had pre-existing HF. In total, the derivation cohort and the validation cohort included the data for 2,383 and 2,011 patients, respectively. The patients’ characteristics in the derivation and validation cohorts are presented in Table 1. Patients in the derivation cohort had a lower prevalence of paroxysmal AF and dyslipidemia and had a higher prevalence of pre-existing HF, hypertension, diabetes mellitus, and chronic kidney disease (all *P* < 0.05). Oral anticoagulants were less frequently prescribed, and cardiothoracic ratio and LV ejection fraction were lower in patients in the derivation cohort than in those in the validation cohort (all *P* < 0.05) (Table 1).

**Table 1:** Patient Characteristics and Clinical Outcomes

	Derivation Cohort (n = 2,383)	Validation Cohort (n = 2,011)	*P* Value
Baseline characteristics			
Age, y	73.8 ± 10.8	73.3 ± 10.9	0.20
Female	933 (39)	832 (41)	0.13
Body mass index, kg/m^2^	23.2 ± 4.2	23.0 ± 3.8	0.14
Body weight, kg	59.6 ± 13.8	59.2 ± 13.1	0.37
Systolic blood pressure, mmHg	125 ± 18	125 ± 20	0.97
Pulse rate, beats/min	80 ± 17	77 ± 15	<0.001
Paroxysmal AF	1,055 (44)	1,139 (57)	<0.001
Smoking history	969 (54)	522 (41)	<0.001
Medical history			
Pre-existing HF	700 (29)	504 (25)	0.001
History of stroke/SE	502 (21)	381 (19)	0.081
Coronary artery disease	335 (14)	309 (15)	0.22
Valvular heart disease	440 (18)	317 (16)	0.018
Cardiomyopathy	59 (2)	63 (3)	0.19
Hypertension	1,609 (68)	1,164 (58)	<0.001
Dyslipidemia	978 (41)	965 (48)	<0.001
Diabetes mellitus	600 (25)	437 (22)	0.007
Peripheral artery disease	108 (5)	70 (3)	0.078
Chronic kidney disease	943 (40)	640 (32)	<0.001
COPD	132 (6)	100 (5)	0.40
History of major bleeding	104 (4)	94 (5)	0.62
Prescription at baseline			
Oral anticoagulants	1,258 (53)	1,168 (59)	<0.001
Warfarin	893 (38)	896 (45)	<0.001
DOAC	365 (15)	272 (14)	0.13
ACE-I/ARBs	1,133 (48)	814 (41)	<0.001
Beta-blockers	674 (28)	676 (34)	<0.001
Loop diuretics	585 (25)	407 (20)	0.001
Biomarkers			
NT-proBNP, ng/L	799 (295-1,839)	756 (288-1,799)	0.69
BNP, ng/L	180 (60-343)	106 (46-217)	0.002
Calculated CrCl, mL/min	56.9 (40.6-77.0)	58.1 (41.4-76.0)	0.67
Hemoglobin, g/dL	12.8 ± 2.1	13.1 ± 2.0	<0.001
Sodium, mEq/L	140 ± 3	141 ± 3	<0.001
Uric acid, mg/dL	6.1 ± 1.8	5.9 ± 2.1	<0.001
Glucose, mg/dL	119 ± 41	116 ± 41	0.036
Chest x-ray data			
Cardio-thoracic ratio, %	54 ± 7	55 ± 7	<0.001
Echocardiographic data			
LV end-diastolic diameter, mm	46.5 ± 6.5	46.6 ± 6.2	0.57
LV end-systolic diameter, mm	31.0 ± 6.8	30.0 ± 6.7	<0.001
LV ejection fraction, %	61.4 ± 11.8	64.7 ± 11.2	<0.001
LV asynergy	420 (21)	354 (23)	0.11
Left atrial diameter, mm	43.1 ± 8.2	44.0 ± 8.2	0.002
Clinical outcomes			
Hospitalization for HF	378 (16)	228 (11)	<0.001
All-cause death	631 (26)	355 (18)	<0.001
Follow-up period, y	4.0 (2.0-7.0)	5.0 (2.1-7.4)	<0.001

Values are mean ± SD, n (%), or median (IQR).

ACE-I = angiotensin converting enzyme inhibitor; AF = atrial fibrillation; ARB = angiotensin receptor blocker; BNP = B-type natriuretic peptide; COPD = chronic obstructive pulmonary disease; CrCl = creatinine clearance; DOAC = direct oral anticoagulants; HF = heart failure; LV = left ventricular; NT-proBNP = N-terminal pro B-type natriuretic peptide; SE = systemic embolism.

### Clinical outcomes

During a median follow-up period of 4.4 years (IQR: 2.1-7.0 years), a total of 606 (14%) hospitalizations for HF occurred among the entire 4,394 patients at a rate of 3.3% per person-year. The Kaplan-Meier curve for the incidence of HF hospitalization is shown in Supplemental Figure 1. The annual incidence rate of HF hospitalization in the derivation cohort was 4.0% per person-year, and that in the validation cohort was 2.5% per person-year. All-cause death occurred in 986 (22%) of 4,394 patients, and the annual mortality rate was 6.1% in the derivation cohort and 3.7% in the validation cohort.

### Performance of ML model

**Figure 1:**
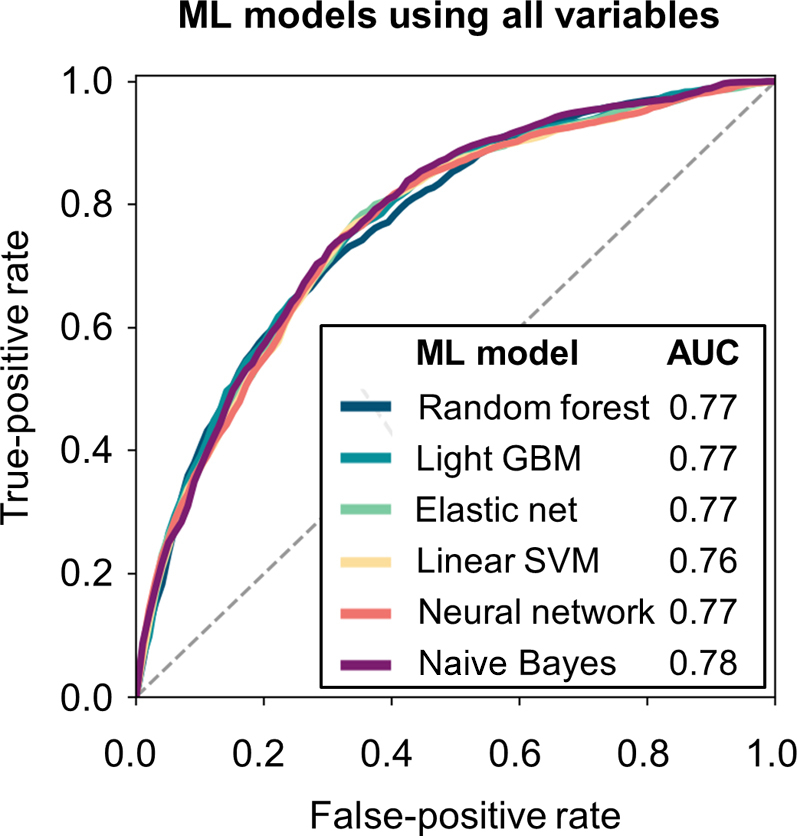
ROC Curves of 6 ML Models Using All Variables Predictive performance of ML models using all 66 variables was examined for the validation cohort. AUC = area under the curve; GBM = gradient boosting machine; ML = machine learning; ROC = receiver operating characteristic; SVM = support vector machine.

The algorithm performance (sensitivity, specificity, accuracy, and AUC) of each ML model using all variables in the derivation cohort are presented in Supplemental Table 3. All 6 models had comparable high predictive performance (AUC range: 0.77-0.83). The performance metrics of the 6 ML models using the validation cohort are shown in Table 2 and Figure 1. Briefly, each ML model had high sensitivity, specificity, and accuracy. The AUCs for each model were also high (range: 0.76-0.78) using the validation cohort.

**Table 2:** Performances of 6 Machine Learning Models Using All Variables in the Validation Cohort

Machine Learning Model	Sensitivity	Specificity	Accuracy	AUC
Random forest	65.8 ± 1.2	73.0 ± 0.4	72.0 ± 0.3	0.77 ± 0.00
Light gradient boosting machine	67.1 ± 1.1	72.7 ± 0.5	72.0 ± 0.3	0.77 ± 0.01
Elastic net	68.6 ± 1.5	71.6 ± 0.6	71.4 ± 0.6	0.77 ± 0.01
Linear support vector machine	68.0 ± 1.0	72.3 ± 0.5	71.7 ± 0.4	0.76 ± 0.00
Neural network	59.6 ± 1.4	77.2 ± 0.4	75.2 ± 0.4	0.77 ± 0.01
Naive Bayes	57.2 ± 1.4	80.1 ± 0.4	77.4 ± 0.5	0.78 ± 0.00

AUC = area under the curve.

### Important variables of each ML model

**Figure 2:**
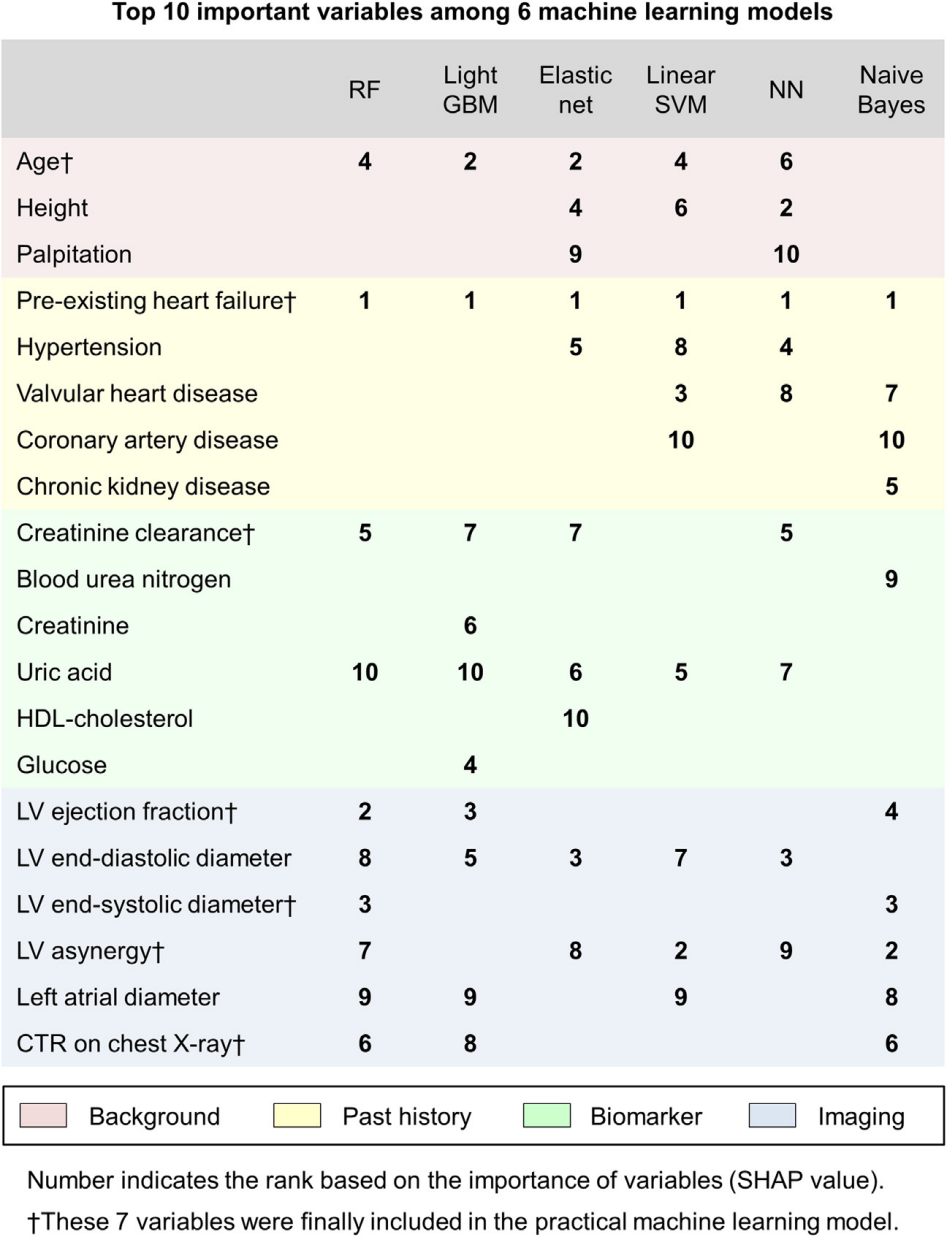
Top 10 Important Variables According to the SHAP Value Variables are color-coded based on the type of variables (**red**, background; **yellow**, past history; **green**, biomarker and **blue**; imaging data). CTR = cardiothoracic ratio; GBM = gradient boosting machine; HDL = high-density lipoprotein; LV = left ventricular; NN = neural network; RF = random forest; SHAP = Shapley Additive exPlanation; SVM = support vector machine.

After calculation of the importance of each variable, the top 10 important variables in each ML model are shown in Figure 2. Pre-existing HF was the most important variable across all 6 ML models. Transthoracic echocardiography data, such as LV ejection fraction, LV diameter, presence of LV asynergy, and left atrial diameter, were frequently included in the top 10 variables in each model. Age, uric acid, and renal function, represented by creatinine clearance, blood urea nitrogen, creatinine, and history of chronic kidney disease were in the top 10 variables in almost all models. Other histories that are common causes of HF, such as hypertension, valvular heart disease, and coronary artery disease, were also included in several ML models.

### Practical risk prediction model using ML algorithms

We extracted the top 7 variables in the random forest algorithm based on their clinical validity, feasibility, and applicability (age, pre-existing HF, LV ejection fraction, LV end-systolic diameter, LV asynergy, creatinine clearance, and cardiothoracic ratio on chest x-ray) (Figure 2).

**Figure 3:**
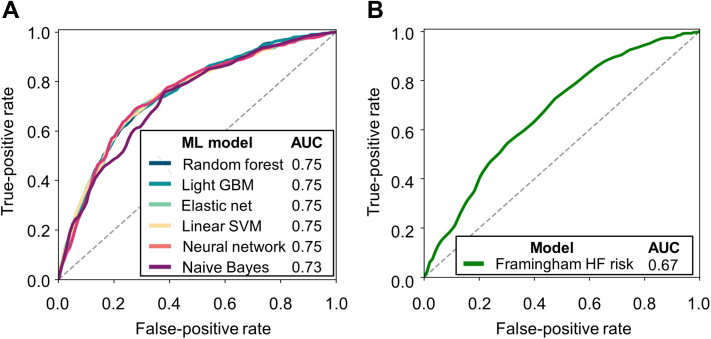
ROC Curves of the Risk Models for the Validation Cohort **(A)** Practical ML models using 7 variables. **(B)** Framingham HF risk model. The 7 variables include age, pre-existing HF, creatinine clearance, cardiothoracic ratio on x-ray, LV ejection fraction, LV end-systolic diameter, and LV asynergy. AUC = area under the curve; GBM = gradient boosting machine; HF = heart failure; LV = left ventricular; ML = machine learning; ROC = receiver operating characteristic; SVM = support vector machine.

The predictive performances of each practical ML model using the 7 variables for the validation cohort are shown in Table 3. The AUCs of the 6 ML algorithms using these 7 variables for the validation cohort are shown in Figure 3A. The AUCs for each model were high (range: 0.73-0.75) using the validation cohort. The AUC of the Framingham HF risk model for the validation cohort is shown in Figure 3B. According to the Hanley and McNeil method, the ML model with random forest algorithm using the 7 variables was significantly superior to the Framingham HF risk model (AUC: 0.75 vs. 0.67; *P* < 0.001).

**Table 3:** Performances of 6 Machine Learning Models Using 7 Variables in the Validation Cohort

Machine Learning Model	Sensitivity	Specificity	Accuracy	AUC
Random forest	67.2 ± 1.6	71.3 ± 1.1	71.0 ± 0.4	0.75 ± 0.01
Light gradient boosting machine	72.0 ± 1.0	65.0 ± 0.6	65.9 ± 0.4	0.75 ± 0.00
Elastic net	68.0 ± 1.0	71.7 ± 0.5	71.0 ± 0.6	0.75 ± 0.01
Linear support vector machine	68.1 ± 1.0	71.7 ± 0.5	71.1 ± 0.6	0.75 ± 0.01
Neural network	68.9 ± 1.1	72.0 ± 0.5	71.7 ± 0.5	0.75 ± 0.01
Naive Bayes	35.8 ± 1.0	89.0 ± 0.4	83.0 ± 0.4	0.73 ± 0.01

The 7 variables include age, pre-existing heart failure, creatinine clearance, cardiothoracic ratio on chest x-ray, left ventricular ejection fraction, left ventricular end-systolic diameter, and left ventricular asynergy.

AUC = area under the curve.

**Figure 4:**
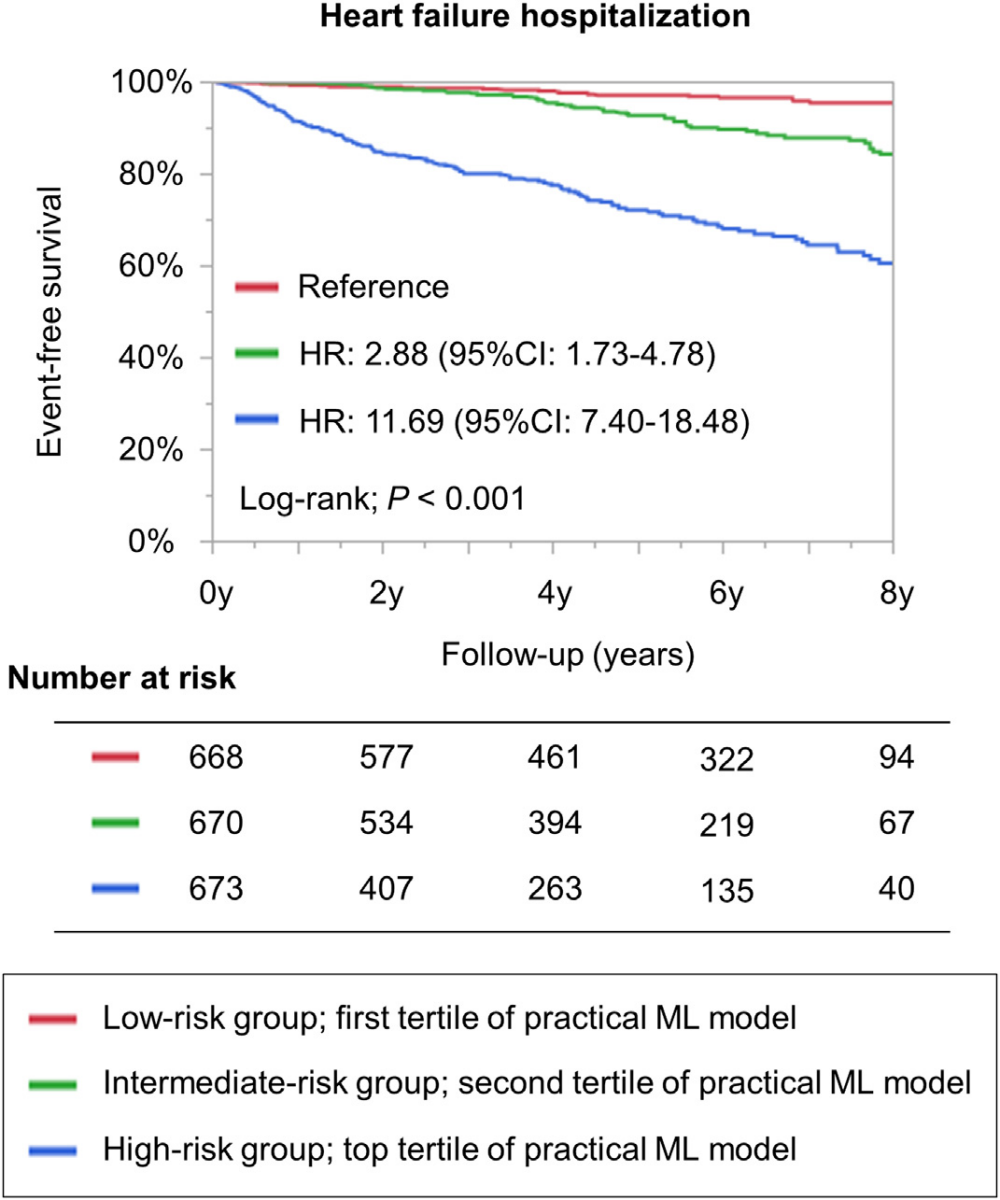
Kaplan-Meier Curves Stratified by Random Forest Algorithm Using 7 Variables The 7 variables include age, pre-existing HF, creatinine clearance, cardiothoracic ratio on x-ray, LV ejection fraction, LV end-systolic diameter, and LV asynergy. CI = confidence interval; HF = heart failure; HR = hazard ratio; ML = machine learning; LV = left ventricular.

The patients’ characteristics stratified by the tertiles of a random forest algorithm using the 7 variables for the validation cohort are presented in Supplemental Table 4. The practical ML risk prediction model was able to stratify the risk of HF hospitalization during the follow-up period (log-rank; *P* < 0.001) (Figure 4). Cox regression analysis revealed that high-risk patients had a 12-fold higher incidence of HF hospitalization during the follow-up period than did low-risk patients (HR: 11.69; 95% CI: 7.40-18.48; *P* < 0.001).

### Among patients without pre-existing HF

**Figure 5:**
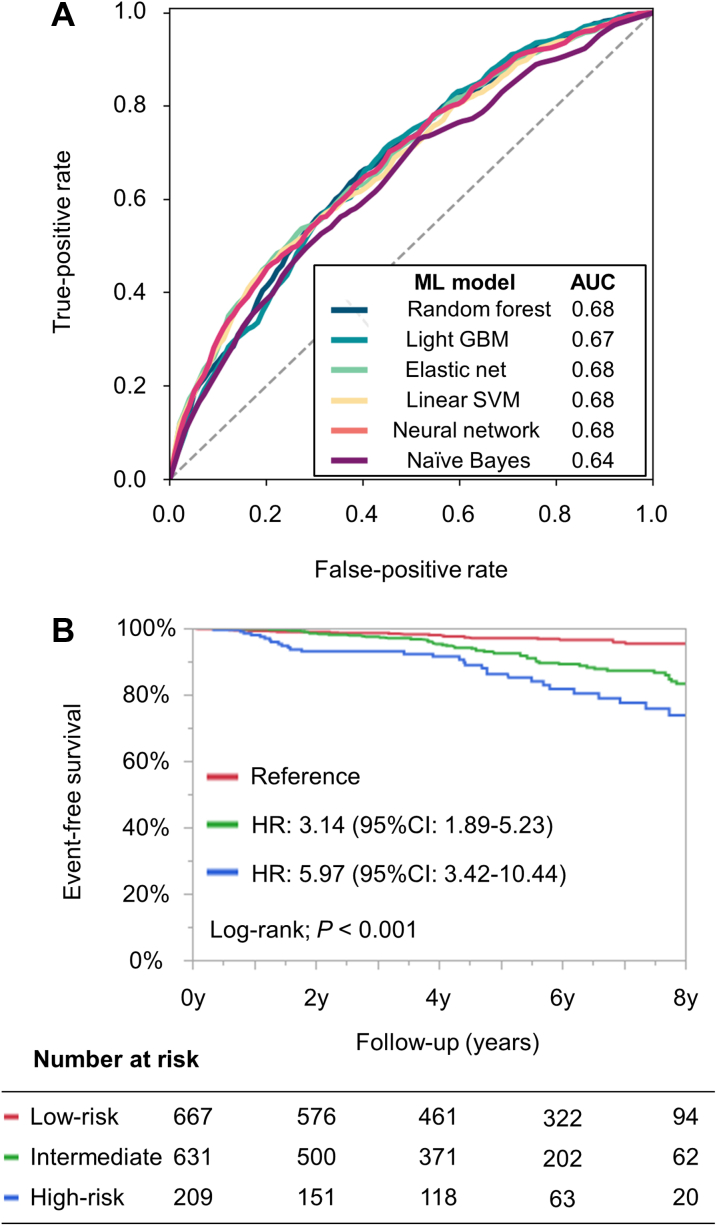
Performance of Practical ML Model Among Patients Without Pre-Existing HF **(A)** ROC curves of the practical ML models. **(B)** Kaplan-Meier curves for the incidence of HF hospitalization stratified by the practical ML model. AUC = area under the curve; CI = confidence interval; GBM = gradient boosting machine; HF = heart failure; HR = hazard ratio; ML = machine learning; ROC = receiver operating characteristic; SVM = support vector machine.

Of 2,011 patients in the validation cohort, 1,507 patients did not have pre-existing HF. Even among patients without pre-existing HF, the practical ML model had a certain level of predictive ability (Figure 5A, Supplemental Table 5). The practical ML model was able to stratify the risk of HF hospitalization among patients without pre-existing HF (log-rank; *P* < 0.001) (Figure 5B). High-risk patients had a 6-fold higher risk (HR: 5.97; 95% CI: 3.42-10.44; *P* < 0.001), and intermediate risk patients had a 3-fold higher risk of HF hospitalization (HR: 3.14; 95% CI: 1.89-5.23; *P* < 0.001) than did low-risk patients.

## Discussion

In the present study, we explored the risk factors and prediction model using ML techniques, and we revealed the following: First, we demonstrated that ML models have a high predictive performance for the incidence of HF hospitalization in patients with AF. Second, transthoracic echocardiographic data and biomarkers were important variables for predicting future HF events. Third, the practical ML model using simple and readily available variables showed a higher predictive ability than did the pre-existing HF risk model and was able to stratify the risk of HF hospitalization among patients with AF (Central Illustration).

**Central Illustration:**
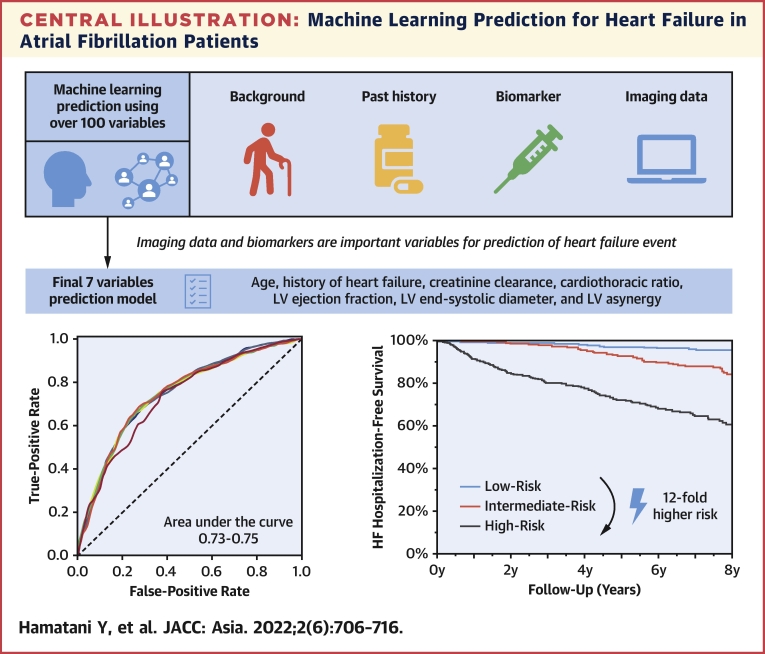
Machine Learning Prediction for Heart Failure in Atrial Fibrillation Patients Machine learning prediction model was created using over 100 variables included in the data set of the Fushimi AF Registry. The entire cohort data were divided into derivation cohort and validation cohort. Imaging data and biomarkers were nominated as important variables for the prediction of future heart failure events. Finally, 7 variables were selected for practical machine learning model based on validity, feasibility, and applicability from the clinician’s perspective. Our practical machine learning model had a certain level of predictive ability and was able to stratify the risk of hospitalization for heart failure in patients with atrial fibrillation. HF = heart failure; LV = left ventricular.

### Risk prediction for incident HF in patients with AF

AF and HF are closely linked and often develop concurrently, with each disease predisposing patients to the other. HF now represents the most common cardiovascular complication in patients with AF, developing at a rate nearly twice that of stroke.^2^^,^^3^ Of note, the incidence of HF did not significantly change over a period of decades despite significant advances in the treatment of patients with AF.^22^^,^^23^ HF accounted for approximately 15% of all-cause mortality among patients with AF in the modern anticoagulation era, which far exceeds that of death due to stroke.^4^^,^^5^ In addition to being frequent, incident HF is associated with a high mortality. Once patients with AF experience HF, they have a risk of mortality that is approximately 2- to 3-fold higher than that of those without.^2^^,^^22^^,^^24^, ^25^, ^26^ These findings underscore the importance of risk stratification for, and prevention of, incident HF in patients with AF.

Several studies have evaluated the significant predictors of incident HF among patients with AF. The ORBIT-AF (Outcomes Registry for Better Informed Treatment of Atrial Fibrillation) reported that significant predictors for incident HF were advanced age, coronary artery disease, valvular heart disease, renal dysfunction, heart rate, and permanent type of AF.^25^ In the United States Woman’s Health Study, well-established modifiable HF risk factors, such as diabetes mellitus and body mass index, were independently associated with the increased risk of HF development in AF.^26^ A few risk prediction scores for incident HF have also been investigated; however, these previous studies used simple standard statistical models like Cox regression analysis with inherent limitations, including correlation between variables, nonlinearity of variables, and limit of the variable number included in the model.^27^^,^^28^ By contrast, ML techniques can overcome these limitations. All ML risk prediction models evaluated in this study had a high predictive performance (AUC: 0.76-0.78), as shown in Table 2 and Figure 1, for predicting hospitalization for HF. ML techniques are expected to be the basis of risk stratification for future HF events in patients with AF.

### Biomarker and echocardiography for risk prediction of HF

This study was unique in that it clarified predictors among data comprising >100 variables, including biological data, histories, biomarkers, and imaging data, using ML algorithms. ML provides the opportunity of discovering new predictors that are not hypothesis driven and without prior assumptions. Previous studies mainly included variables related to the patients’ backgrounds and comorbid conditions, and they were unable to address the importance of biomarkers and imaging data.^25^^,^^26^^,^^29^ Of note, our ML model suggested that imaging data and biomarkers are important variables for predicting incident HF, revealed by their prominent presence on the list of top 10 variables shown in Figure 2.

Indeed, the Belgrade AF Study reported that mild left atrial dilatation or low-normal LV ejection fraction in structurally normal heart heralds an increased risk of incident HF.^30^ Another study reported that increased left atrial volume provided prognostic information for the prediction of HF events in AF.^31^ When these previous studies are combined with ours, transthoracic echocardiography plays an important role in risk stratification for incident HF in patients with AF. In addition to imaging data, our study suggested that biomarkers can help identify patients with AF who are at an increased risk of HF events. We previously demonstrated that natriuretic peptide levels are a useful biomarker for the risk stratification of HF hospitalization in patients with AF, although this biomarker was unable to be included in our ML models because of missing data.^32^ Biomarkers of inflammation, kidney function, and hemoglobin levels were also reportedly associated with a higher incidence of HF in these patients.^28^^,^^33^^,^^34^ However, there is a scarcity of studies incorporating imaging data and biomarkers for the risk prediction model of HF events. Our ML models using these imaging and biomarker data had a high predictive ability, which suggests the utility of incorporating these data for risk stratification for incident HF in patients with AF.

### Implication of ML risk prediction models in clinical practice

Some burdens are specific to the application of ML models in daily practice. In particular, risk stratification using dozens of variables is difficult or almost impossible to implement in clinical practice. Therefore, we ultimately selected several variables for risk prediction with a view to their future practicality. A practical ML model incorporating only 7 variables (age, pre-existing HF, renal function, cardiothoracic ratio, and echocardiographic LV parameters) has the potential to become an appropriate risk prediction tool for future HF events among patients with AF. Objective data for these 7 variables are easy to obtain, and we believe that our ML model can be readily available in clinical practice. Of note, a random forest model using these 7 variables was shown to have a higher predictive ability than the Framingham HF risk model (Figure 3). Even among patients without pre-existing HF, our practical ML model was able to stratify the risk of HF hospitalization and had a certain level of predictive ability, albeit numerically lower than that among the entire cohort.

Recently, catheter ablation or pharmacological therapy, including sodium glucose co-transporter 2 inhibitors, was reported to aid in preventing HF development in selected patients with AF.^35^^,^^36^ However, it may not be practical to give these therapies to all patients with AF, considering the inherent complications, procedural costs, and large target number. To effectively prevent the development of HF in patients with AF, it is important to identify high-risk patients as a first step. By establishing a risk prediction model using techniques like ML algorithms, studies addressing whether these interventions can prevent the incidence of HF in high-risk patients are warranted in the future.

### Study limitations

The present study has several limitations. First, this was an observational study and provides only associative evidence, not causative. Second, some potential important variables were excluded because of a large number of missing values. Indeed, two thirds of patients without pre-existing HF had no data for natriuretic peptide levels in the registry. However, we specifically clarified their prognostic significance for future HF events, highlighting the importance of measuring natriuretic peptide levels in all patients with AF.^32^ In addition, we did not collect detailed echocardiographic data, including diastolic dysfunction and biomarkers such as troponin levels in this registry. We may further increase the predictive ability of the risk algorithm for incident HF with additional measures. Third, even though we show the predictive ability of clinically available 7 variables, it is plausible that the optimal number of variables is different. In addition, chest x-ray and echocardiography might not be available for all patients with AF, especially in primary care clinics. Fourth, ML models are at high risk of overfitting, and overfitting can be truly assessed only in external data. Although our ML model demonstrated good discrimination ability in the registry, external validation is strongly warranted. Inasmuch as we used a single community-based registry, external sampling in a completely separate population is desirable. Fifth, we did not obtain echocardiographic data at HF hospitalization, and incident HF could not be classified according to LV ejection fraction. Considering these limitations, further studies are warranted to create more accurate ML risk prediction models incorporating additional important variables with external validation. We hope that our study, which suggests the utility of the ML model, forms a foundation for the prediction of HF events in patients with AF.

## Conclusions

ML algorithms had a high predictive performance for HF hospitalization in patients with AF. Imaging data and biomarkers were important variables across all ML models, which suggests their utility for risk prediction of HF events. Our ML model using 7 simple and readily available variables was able to stratify the risk of hospitalization for HF in patients with AF, providing opportunities for the implementation of strategies to prevent HF among patients with AF.

Perspectives **COMPETENCY IN MEDICAL KNOWLEDGE:** ML revealed that the data of transthoracic echocardiography and biomarkers were important predictors for HF hospitalization in patients with AF. The ML model incorporating these several variables can stratify the risk of HF among patients with AF in daily practice.

**TRANSLATIONAL OUTLOOK:** Further studies are needed to create more accurate risk prediction models incorporating additional important variables, and to investigate the efficacy of pharmacological and/or non-pharmacological therapies for preventing HF events in high-risk patients with AF.

## Funding Support and Author Disclosures

The Fushimi AF Registry is supported by research funding from Boehringer Ingelheim, Bayer Healthcare, Pfizer, Bristol-Myers Squibb, Astellas Pharma, AstraZeneca, Daiichi Sankyo, Novartis Pharma, MSD, Sanofi-Aventis, and Takeda Pharmaceutical. The sponsors had no role in the design or conduct of the study; collection, management, analysis, and interpretation of the data; or preparation, review, or approval of the manuscript. This study was partially supported by the Practical Research Project for Life-Style related Diseases including Cardiovascular Diseases and Diabetes Mellitus from Japan Agency for Medical Research and Development, AMED (19ek0210082h0003, 18ek0210056h0003). Dr Akao has received lecture fees from Pfizer, Bristol-Myers Squibb, Boehringer Ingelheim, Bayer Healthcare, and Daiichi-Sankyo. All other authors have reported that they have no relationships relevant to the contents of this paper to disclose.
