# Supplementary material for: Machine Learning Risk Prediction for Incident Heart Failure in Patients With Atrial Fibrillation
Source: JACC Asia. 2022 Nov 1;2(6):706–16. doi: 10.1016/j.jacasi.2022.07.007 (PMC9700042; doi:10.1016/j.jacasi.2022.07.007)
Supplement: Supplemental Figure 1 and Tables 1–5 [file mmc1.docx]

**Machine Learning Risk Prediction for Incident Heart Failure in Patients with Atrial Fibrillation**

Yasuhiro Hamatani, MD, PhD,^a^ Hidehisa Nishi, MD, PhD,^b^ Moritake Iguchi, MD, PhD,^a^ Masahiro Esato, MD, PhD,^c^ Hikari Tsuji, MD, PhD,^d^ Hiromichi Wada, MD, PhD,^e^ Koji Hasegawa, MD, PhD,^e^ Hisashi Ogawa, MD,^a^ Mitsuru Abe, MD, PhD,^a^ Shunichi Fukuda, MD, PhD,^f^ and Masaharu Akao, MD, PhD,^a^ on behalf of the Fushimi AF Registry investigators

**SUPPLEMENTAL APPENDIX**

**Machine Learning Risk Prediction for Incident Heart Failure in Patients with Atrial Fibrillation**

| **Supplemental Table 1.** Variables for creating machine learning models, and number and percentage of missing data in the derivation cohort | | |
| --- | --- | --- |
| Variables | Number of missing data | % of missing data |
| Age | 0 | 0.0 |
| Sex | 0 | 0.0 |
| Height | 194 | 8.1 |
| Weight | 119 | 5.0 |
| Body mass index | 202 | 8.5 |
| Body surface area | 202 | 8.5 |
| Systolic blood pressure | 4 | 0.2 |
| Diastolic blood pressure | 4 | 0.2 |
| Pulse rate | 6 | 0.3 |
| Baseline cardiac rhythm on electrocardiography | 0 | 0.0 |
| Type of AF | 0 | 0.0 |
| Valvular AF | 0 | 0.0 |
| AF duration | 527 | 22.1 |
| Palpitation | 0 | 0.0 |
| Shortness of breath | 0 | 0.0 |
| Fatigue | 0 | 0.0 |
| Chest pain | 30 | 1.3 |
| Dizziness | 30 | 1.3 |
| Previous stroke | 0 | 0.0 |
| Previous cerebral infarction | 0 | 0.0 |
| Previous intracranial hemorrhage | 0 | 0.0 |
| Previous systemic thromboembolism | 0 | 0.0 |
| Previous transient ischemic attack | 0 | 0.0 |
| Previous major bleeding | 0 | 0.0 |
| Pre-existing heart failure | 0 | 0.0 |
| Valvular heart disease | 0 | 0.0 |
| Mitral regurgitation | 0 | 0.0 |
| Aortic regurgitation | 0 | 0.0 |
| Aortic stenosis | 0 | 0.0 |
| Tricuspid regurgitation | 0 | 0.0 |
| Surgical history for valvular heart disease | 0 | 0.0 |
| Hypertrophic cardiomyopathy | 0 | 0.0 |
| Dilated cardiomyopathy | 0 | 0.0 |
| Hypertension | 0 | 0.0 |
| Diabetes mellitus | 0 | 0.0 |
| Dyslipidemia | 0 | 0.0 |
| Coronary artery disease | 0 | 0.0 |
| Previous myocardial infarction | 0 | 0.0 |
| Previous percutaneous coronary intervention | 0 | 0.0 |
| Previous coronary artery bypass grafting | 0 | 0.0 |
| Peripheral artery disease | 0 | 0.0 |
| Chronic kidney disease | 1 | 0.1 |
| Hemodialysis | 0 | 0.0 |
| Chronic obstructive pulmonary disease | 0 | 0.0 |
| Pacemaker implantation | 0 | 0.0 |
| Implantable cardioverter defibrillator | 0 | 0.0 |
| Cardiac resynchronization therapy | 0 | 0.0 |
| History of cardiac ablation | 0 | 0.0 |
| Hemoglobin | 64 | 3.0 |
| Sodium | 281 | 11.8 |
| Creatine phosphokinase | 495 | 20.8 |
| Creatinine | 63 | 2.6 |
| Blood urea nitrogen | 84 | 3.5 |
| Creatinine clearance | 174 | 7.3 |
| Uric acid | 690 | 28.9 |
| Triglyceride | 345 | 14.5 |
| High-density lipoprotein cholesterol | 543 | 22.8 |
| Low-density lipoprotein cholesterol | 533 | 22.4 |
| Glucose | 218 | 9.1 |
| Cardio-thoracic ratio on chest X-ray | 244 | 10.2 |
| Left ventricular end-diastolic diameter | 417 | 17.4 |
| Left ventricular end-systolic diameter | 433 | 18.2 |
| Left ventricular ejection fraction | 400 | 16.8 |
| Left atrial diameter | 425 | 17.8 |
| Relative wall thickness | 434 | 18.2 |
| Left ventricular wall motion asynergy | 379 | 15.9 |

AF = atrial fibrillation.

| **Supplemental Table 2.** Hyperparameters of the machine learning model | | |
| --- | --- | --- |
| Model | Hyperparameter | Searched value or category |
| LGBM | Lambda l1 | 1e-8,1e-7,1e-6,1e-5,1e-4,1e-3,1e-2,1e-1,1,10 |
|  | Lambda l2 | 1e-8,1e-7,1e-6,1e-5,1e-4,1e-3,1e-2,1e-1,1,10 |
|  | Number of leaves | 2­­­–256 |
|  | Feature fraction | 0.4, 0.5, 0.6, 0.7, 0.8, 0.9, 1.0 |
|  | Bagging fraction | 0.4, 0.5, 0.6, 0.7, 0.8, 0.9, 1.0 |
|  | Bagging frequency | 1­–20 |
|  | Minimum child samples | 5­­­–100 |
|  | Max depth | 2­–50 |
| RF | Number of estimators | 2000 |
|  | Criterion of impurity | Gini impurity, entropy |
|  | Maximum features | 3—20 |
|  | Max depth of trees | 2—15 |
|  | Max number of leaf nodes | 2—100 |
|  | Minimum impurity decrease | 0—1 |
|  | Minimum samples of leaf | 1—100 |
|  | Minimum samples to split | 7—100 |
| RLR | L1 ratio | 0, 0.1, 0.2, 0.3, 0.4, 0.5, 0.6, 0.7, 0.8, 0.9, 1.0 |
|  | C | 1e-8,1e-7,1e-6,1e-5,1e-4,1e-3,1e-2,1e-1,1,10 |
| SVM | Gamma | 1e-8,1e-7,1e-6,1e-5,1e-4,1e-3,1e-2,1e-1,1,10 |
|  | C | 1e-8,1e-7,1e-6,1e-5,1e-4,1e-3,1e-2,1e-1,1,10 |
| MLP | Hidden layer sizes | 2­–100 |
|  | Activation | identity, logistic, tanh, relu |
|  | Solver | lbfgs, sgd, adam |
|  | Alpha | 1e-8,1e-7,1e-6,1e-5,1e-4,1e-3,1e-2,1e-1,1,10 |
|  | Initial learning rate | 1e-8,1e-7,1e-6,1e-5,1e-4, 1e-3, 1e-2 |
|  | Max iteration | 100–1000 |
|  | tolerance | 1e-8,1e-7,1e-6,1e-5,1e-4 |

MLP = multilayer perceptron; LGBM = light gradient boosting machine; RF = random forest; RLR = regularized logistic regression; SVM = support vector machine.

| **Supplemental Table 3.** Performances of 6 machine learning models in the derivation cohort | | | | |
| --- | --- | --- | --- | --- |
| Machine Learning Model | **Sensitivity** | **Specificity** | **Accuracy** | **AUC** |
| Random forest | 74 | 76 | 75 | 0.83 |
| Light gradient boosting machine | 71 | 75 | 75 | 0.80 |
| Elastic net | 70 | 74 | 73 | 0.78 |
| Linear support vector machine | 69 | 74 | 73 | 0.78 |
| Neural network | 62 | 79 | 80 | 0.78 |
| Naïve Bayes | 55 | 83 | 78 | 0.77 |

AUC = area under the curve.

| **Supplemental Table 4.** Patient characteristics stratified by the tertiles of practical ML model | | | | |
| --- | --- | --- | --- | --- |
|  | **Low-risk** | **Intermediate-risk** | **High-risk** | *P*-value |
|  | (n = 668) | (n = 670) | (n = 673) |  |
| Baseline characteristics |  |  |  |  |
| Age, y | 65.5 ± 10.1 | 76.4 ± 8.3 | 78.1 ± 9.7 | <0.001 |
| Female | 199 (30%) | 320 (48%) | 313 (47%) | <0.001 |
| Body mass index, kg/m^2^ | 23.9 ± 3.5 | 22.7 ± 3.6 | 22.5 ± 4.0 | <0.001 |
| Body weight, kg | 64.7 ± 11.4 | 57.1 ± 12.3 | 56.5 ± 13.7 | <0.001 |
| Systolic BP, mmHg | 127 ± 19 | 125 ± 19 | 123 ± 21 | 0.001 |
| Pulse rate, beats/min | 75 ± 13 | 77 ± 15 | 78 ± 16 | 0.001 |
| Paroxysmal AF | 450 (67%) | 405 (60%) | 284 (42%) | <0.001 |
| Smoking history | 198 (49%) | 125 (33%) | 199 (40%) | <0.001 |
| Medical history |  |  |  |  |
| Pre-existing HF | 1 (0.2%) | 39 (6%) | 464 (69%) | <0.001 |
| History of stroke/SE | 98 (15%) | 140 (21%) | 143 (21%) | 0.003 |
| Coronary artery disease | 47 (7%) | 83 (12%) | 179 (27%) | <0.001 |
| Valvular heart disease | 43 (6%) | 86 (13%) | 188 (28%) | <0.001 |
| Cardiomyopathy | 9 (1%) | 18 (3%) | 36 (5%) | <0.001 |
| Hypertension | 354 (53%) | 392 (59%) | 418 (62%) | 0.003 |
| Dyslipidemia | 316 (47%) | 336 (50%) | 313 (47%) | 0.37 |
| Diabetes mellitus | 138 (21%) | 151 (23%) | 148 (22%) | 0.69 |
| Peripheral artery disease | 13 (2%) | 23 (3%) | 34 (5%) | 0.008 |
| Chronic kidney disease | 77 (12%) | 218 (33%) | 345 (51%) | <0.001 |
| COPD | 22 (3%) | 31 (5%) | 47 (7%) | 0.007 |
| History of major bleeding | 23 (3%) | 28 (4%) | 43 (6%) | 0.029 |
| Prescription at baseline |  |  |  |  |
| Oral anticoagulants | 350 (53%) | 387 (58%) | 431 (65%) | <0.001 |
| Warfarin | 251 (38%) | 300 (45%) | 345 (52%) | <0.001 |
| DOAC | 99 (15%) | 87 (13%) | 86 (13%) | 0.49 |
| ACE-I/ARBs | 234 (35%) | 265 (40%) | 315 (47%) | <0.001 |
| β-blockers | 210 (32%) | 177 (27%) | 289 (44%) | <0.001 |
| Loop diuretics | 41 (6%) | 90 (14%) | 276 (42%) | <0.001 |
| Biomarkers |  |  |  |  |
| NT-proBNP, ng/L | 197 (73, 552) | 582 (218, 835) | 1247 (603, 2708) | <0.001 |
| BNP, ng/L | 56 (24, 116) | 94 (54, 194) | 159 (87, 280) | <0.001 |
| Calculated CrCl, mL/min | 77.9 (65.5, 94.7) | 51.7 (40.9, 66.7) | 44.5 (32.2, 62.0) | <0.001 |
| Hemoglobin, g/dL | 14.0 ± 1.5 | 13.0 ± 1.9 | 12.4 ± 2.0 | <0.001 |
| Sodium, mEq/L | 141 ± 2 | 141 ± 3 | 140 ± 3 | 0.002 |
| Uric acid, mg/dL | 5.6 ± 1.6 | 5.7 ± 1.7 | 6.3 ± 2.7 | <0.001 |
| Glucose, mg/dL | 115 ± 41 | 115 ± 37 | 117 ± 45 | 0.48 |
| Chest X-ray data |  |  |  |  |
| Cardio-thoracic ratio, % | 50.1 ± 5.6 | 55.4 ± 6.5 | 57.6 ± 7.3 | <0.001 |
| Echocardiographic data |  |  |  |  |
| LVEDD, mm | 45.7 ± 4.6 | 45.2 ± 5.3 | 48.7 ± 7.6 | <0.001 |
| LVESD, mm | 27.6 ± 3.6 | 27.9 ± 4.1 | 34.1 ± 8.7 | <0.001 |
| LV ejection fraction, % | 69.7 ± 5.4 | 68.3 ± 6.3 | 56.5 ± 13.9 | <0.001 |
| LV asynergy | 6 (1%) | 47 (10%) | 301 (57%) | <0.001 |
| Left atrial diameter, mm | 41.4 ± 7.0 | 43.4 ± 7.3 | 47.0 ± 9.0 | <0.001 |
| Values are the mean ± SD, median (interquartile range) or n (%). ACE-I = angiotensin converting enzyme inhibitor; AF = atrial fibrillation; ARB = angiotensin receptor blocker; BNP = B-type natriuretic peptide; BP = blood pressure; COPD = chronic obstructive pulmonary disease; CrCl = creatinine clearance; DOAC = direct oral anticoagulants; HF = heart failure; LV = left ventricular; LVEDD = left ventricular end-diastolic diameter; LVESD = left ventricular end-systolic diameter; ML = machine learning; NT-proBNP = N-terminal pro B-type natriuretic peptide; SE = systemic embolism. | | | | |

| **Supplemental Table 5.** Performances of practical machine learning models among patients without pre-existing HF in the validation cohort. | | | | |
| --- | --- | --- | --- | --- |
| Machine Learning Model | Sensitivity | Specificity | Accuracy | AUC |
| Random forest | 36.2 ± 1.5 | 82.6 ± 0.5 | 78.9 ± 0.3 | 0.68 ± 0.01 |
| Light gradient boosting machine | 49.2 ± 0.9 | 73.0 ± 0.5 | 71.0 ± 0.4 | 0.67 ± 0.00 |
| Elastic net | 54.3 ± 1.3 | 71.5 ± 0.5 | 70.1 ± 0.2 | 0.68 ± 0.01 |
| Linear support vector machine | 36.6 ± 1.1 | 86.0 ± 0.2 | 82.0 ± 0.2 | 0.68 ± 0.01 |
| Neural network | 51.4 ± 1.0 | 72.8 ± 0.4 | 71.0 ± 0.2 | 0.68 ± 0.01 |
| Naïve Bayes | 8.5 ± 0.9 | 97.3 ± 0.5 | 90.0 ± 0.0 | 0.64 ± 0.01 |
| AUC = area under the curve; HF = heart failure. | | | | |

**Supplemental Figure 1**


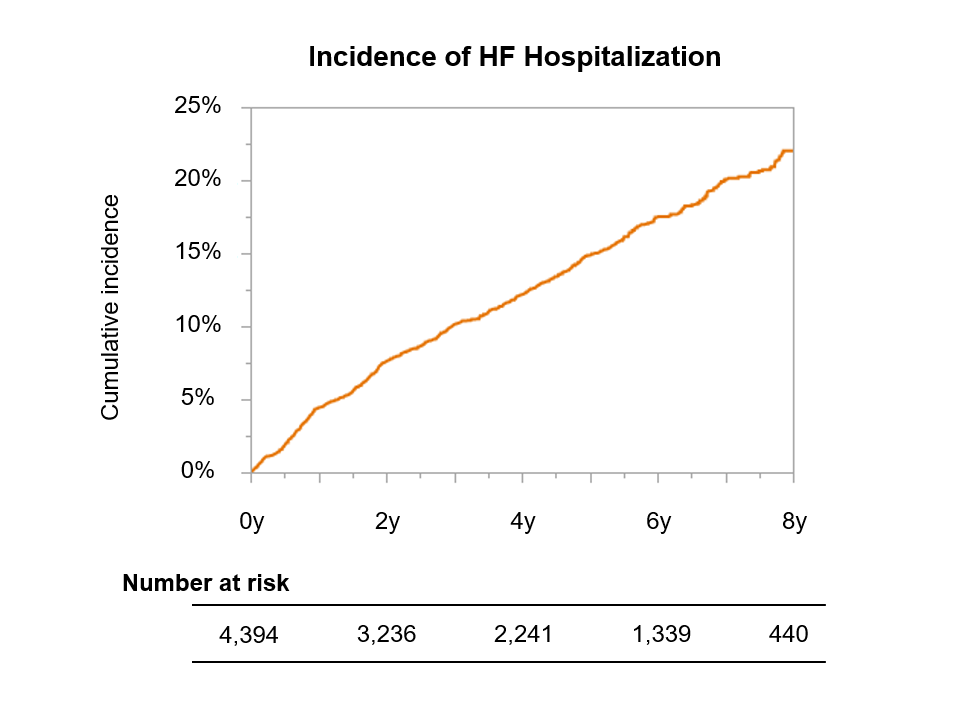


**Figure legend:**

Kaplan-Meier curve for the incidence of HF hospitalization during the follow-up period in the Fushimi AF Registry.

AF = atrial fibrillation; HF = heart failure.
